# Effects of Different Sulfur Compounds on the Distribution Characteristics of Subcellular Lead Content in *Arabis alpina* L. var. *parviflora* Franch under Lead Stress

**DOI:** 10.3390/plants12040874

**Published:** 2023-02-15

**Authors:** Cui Xu, Li Qin, Yuan Li, Yanqun Zu, Jixiu Wang

**Affiliations:** College of Resources and Environment, Yunnan Agricultural University, Kunming 650201, China

**Keywords:** *A. alpina*, sulfur, lead, cell walls, soluble fraction

## Abstract

Sulfur plays a vital role in the phytoremediation of lead-contaminated soil. The effects of different sulfur forms (S Na_2_S, and Na_2_SO_4_) on lead (Pb) absorption in hyperaccumulator *Arabis alpina* L. var. *parviflora* Franch were studied in a soil pot experiment. The subcellular sulfur and lead enrichment characteristics in *A. alpina* were studied by adding sulfur in different forms and concentrations (0, 75, and 150 mg·kg^−1^) to Pb-contaminated soil. The results show that the root and shoot biomass increased by 1.94 times under Na_2_S and Na_2_SO_4_ treatment, and the root–shoot ratio of *A. alpina* increased 1.62 times under the three forms of sulfur treatments, compared with the control. Sulfur content in cell walls and soluble fractions of the root and shoot of *A. alpina* significantly increased 3.35~5.75 times and decreased 5.85 and 9.28 times in the organelles under 150 mg·kg^−1^ Na_2_SO_4_ treatment. Meanwhile, Pb content in the root and shoot cell walls of *A. alpina* significantly increased by 3.54 and 2.75 times, respectively. Pb content in the shoot soluble fraction increased by 3.46 times, while it significantly reduced by 3.78 times in the shoot organelle. Pb content in the root organelle and soluble fraction decreased by 2.72 and 2.46 times. Different forms and concentrations of sulfur had no regularity in the effect of Pb and sulfur content in the subcellular components of *A. alpina*, but the bioconcentration and translocation factors of *A. alpina* increased compared with the control. Under different concentrations of Na_2_SO_4_, there was a significant positive correlation between the contents of sulfur and Pb in the subcellular components of the root of *A. alpina* (*p* < 0.05). These results indicate that sulfur application can enhance the Pb resistance of *A. alpina* by strengthening the cell wall fixation and vacuolar compartmentalization.

## 1. Introduction

Lead has a relatively large molecular weight and migrates slowly, so its chemical cycling in the soil is mainly influenced by human factors. The analysis of lead pollution sources in the environment also confirmed that industry and agriculture are major contributors [1]. Lead migrates from soil to plants and accumulates in organisms, and eventually endangers human health through the food chain. Many factors affecting the migration and transformation of lead in soil are reported, such as the form of lead, soil type, soil properties, soil nutrients, and fertilization [2]. Sulfur application can change the growth condition of crops and promote soil remediation or reduce pollution as a non-metallic element, which positively affects plant adsorption of heavy metals [3]. The cyclic transformation of sulfur in the soil can significantly affect heavy metals’ biogeochemical process and play an essential biological role in the plant’s physiological metabolism against heavy metal ion pollution and the detoxification of heavy metals [4]. The current research on the effect of sulfur on heavy metals is contradictory [5]. One view is that sulfur can activate heavy metals in soil and increase their bioavailability. Sulfur application to soil can significantly increase the proportion of soluble copper (Cu) and zinc (Zn) in soil and increase Cu accumulation in roots and shoots of *Elsholtzia chinensis* [6]. Another view is that sulfur can inactivate heavy metals in soil and reduce their bioavailability. The application of fertilizers containing sulfur to Cu-contaminated grassland could reduce the levels and toxicity of Cu [7]. Studies showed that exogenous sulfur can reduce the absorption and toxicity of heavy metals to plants. Sulfur-impregnated organoclay enhanced the phytoextraction of cadmium (Cd), chromium (Cr), Cu, nickel (Ni), Pb, and Zn by the roots and reduced their translocation into the shoots of pea (*Pisum sativum*) and corn (*Zea mays*) [8]. Application of S reduced the accumulation of Cu in castor (*Ricinus communis* L.) and promoted its growth [9]. Exogenous supplementation of sulfur reduced arsenic (As) toxicity in shoots of *Isatis cappadocica* Desv and *Erysimum allionii* L. [10].

There are many literature reports on the regulatory role of sulfur in the phytoremediation of soil heavy metals [11,12,13]. Exogenous application of sulfur reduces the uptake and toxic effects of heavy metals by plants. The activation or deactivation of heavy metals by sulfur in soil may be related to the form of sulfur. Different sulfur forms and concentrations significantly affected the heavy metals extraction [4].

At the subcellular level, sulfur application increases the proportion of heavy metals in cell walls and vacuolar, while changes in sulfur valence in soil also cause changes in soil pH, which has a direct effect on the dissolved sorption of heavy metals and increases the proportion of heavy metals fixed/deposited by plant roots [14,15]. Furthermore, the sulfur-containing compounds formed by different chelating forms of sulfur into the plant changed the subcellular distribution of lead, and plant cell wall retention and vacuolar compartmentalization play a major regulatory role in the detoxification and tolerance of hyperaccumulator plants to heavy metals [16]. Sulfur addition significantly increases the content of cadmium in the cell wall and soluble fractions of *Southeastern Sedum* [17]. Plants participate in the metabolic processes of cysteine (Cys), methionine (Met), small molecule glutathione (GSH), and phytochelatins (PCs) during the stress response [3]. The sulfhydryl substances in the metabolic process were combined with heavy metals in the cells and then transferred and stored in vacuoles, thereby alleviating the toxic effects of heavy metals on plant [13]. Lin and Shi [18] used K-edge X-ray absorption spectroscopy (XANES) and found that the sulfhydryl functional groups that chelate metals in the soil have significant differences between rhizosphere and non-rhizosphere soils. The K-edge XANES of sulfur indicated that the content of high-valent sulfur in rhizosphere soil was higher than in non-rhizosphere soil, and the valence of heavy metals from rhizosphere soil to plants was also changing, indicating that different forms of S compounds in plants are closely related to detoxification mechanisms.

*A. alpina* as a Zn, Pb, and Cd hyperaccumulator, could be used for long-term phytoremediation of soils contaminated by Cd, Pb, and Zn, Pb contents in shoot of *A. alpina* is more than 1000 mg·kg^−1^, Cd content in shoot >100 mg·kg^−1^ and Zn contents in shoot >10,000 mg·kg^−1^, with the enrichment factors and transport coefficients greater than 1 [19]. Our previous studies showed that, under heavy metal stress, the application of sulfur treatment at sulfur concentrations of 50 and 100 mg·kg^−1^ (as Na_2_SO_4_) increased the activities of related enzymes involved in sulfur-containing compounds in *A.alpina*, including γ-glutamylcysteine synthetase (γ-GCS), Glutathione reductase (GR) and Glutathione S-transferase (GST) activities in the root part of the plant [20]. However, the changes in sulfur compounds and heavy metal content in the different cellular fractions of *A.alpina* plants following S treatment are not known; particularly, the relationship between these changes and the uptake of accumulated heavy metals by the plants is a topic that needs to be investigated. In this paper, pot experiments were conducted to study the shoot and root biomass of *A. alpina* under different sulfur forms and treatments, the accumulation characteristics of lead, and the distribution of lead in subcellular components to reveal the influence of sulfur form on the uptake and enrichment of lead by hyperaccumulator plants. The response mechanism of *A. alpina* to lead stress under the action of sulfur and the mechanism of *A. alpina*-enriched lead at the subcellular level was explored and used to provide theoretical and technical guidance for using hyperaccumulator plants *A. alpina* to remediate lead-contaminated soil.

## 2. Results

### 2.1. The Effect of Different Forms of Sulfur on the Biomass of A. alpina

As shown in Table 1, Na_2_S and Na_2_SO_4_ treatments significantly changed the root and shoot biomass of *A. alpina* compared with the control. Na_2_S treatment at 75 mg·kg^−1^ and 150 mg·kg^−1^ increased the root biomass 1.83 and 2.33 times, Na_2_SO_4_ increased 3.00 and 3.28 times, respectively. Compared with the control (CK), 75 mg·kg^−1^ and 150 mg·kg^−1^ Na_2_SO_4_ treatments significantly increased the shoot biomass of *A. alpina* by 1.59 and 2.01 times. Different forms of exogenous sulfur increased the root-to-shoot ratio of *A. alpina* (except for 75 mg·kg^−1^ elemental sulfur treatment), indicating that sulfur promotes the growth of the root system of *A. alpina*, which is beneficial to increase the contact of *A. alpina* with lead-contaminated soil.

### 2.2. Effects of Different Forms of Sulfur on the Sulfur Contents in Subcellular Part of A. alpina

**Figure 1:**
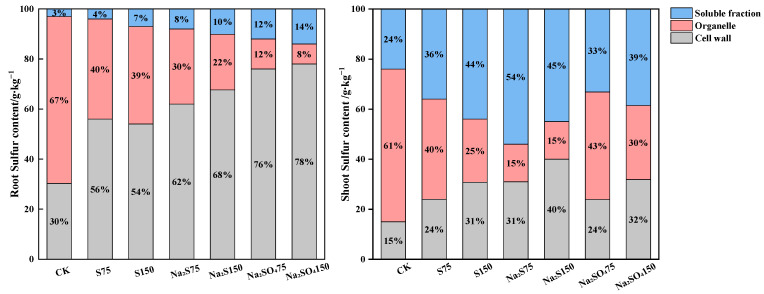
Percentages of subcellular distribution of sulfur in *A. alpina*. The legend indicates sulfur in different subcellular components of *A. alpina*/(g·kg^−1^).

It can be seen from Table 2 that the three sulfur forms significantly changed the S content in the subcellular components of *A. alpina*, but different forms of sulfur had different distributions of sulfur content in the root and shoot cell walls, organelles, and soluble components of *A. alpina*. Compared with the CK, 75 mg·kg^−1^ and 150 mg·kg^−1^ elemental S treatments significantly reduced the sulfur content in the root and shoot organelles of *A. alpina* by 58% and 49%. Na_2_S treatment significantly increased the sulfur content of the root cell wall and the soluble component of *A. alpina* by 116% and 183% and significantly reduced the organelle sulfur content by 68% (Figure 1). Further, the shoot cell wall and soluble component sulfur content increased by 82% and 90%, respectively, and the sulfur content of organelles was significantly reduced by 84%. Na_2_SO_4_ treatment significantly increased the sulfur content of the root and shoot cell walls and soluble components of *A. alpine* by 3.65, 5.75, 4.58, and 3.35 times, respectively, and significantly reduced the organelle sulfur content to 83% and 89%.

### 2.3. Effects of Different Forms of Sulfur on Lead Content in Subcellular Part of A. alpina

**Figure 2:**
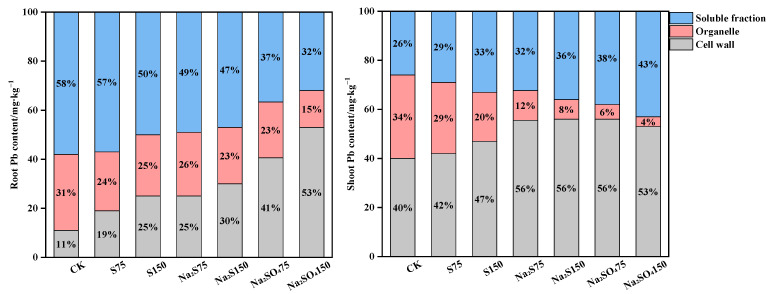
Percentages of subcellular distribution of lead in *A. alpina*. The legend indicates Pb in different subcellular components of *A. alpina*/(mg·kg^−1^).

From Table 3, compared with the CK, it can be seen that different forms of sulfur treatment significantly changed the lead content in the root and shoot subcells of *A. alpina*; in addition, the SO_4_^2−^ treatment, as well as S and Na_2_S treatments increased Pb in the lower ground cell wall by 58% to 101% compared to CK, but there was no significant difference between the two treatments and the different concentrations. Under 150 mg·kg^−1^ Na_2_SO_4_ treatment, the lead content in the root and shoot cell walls of *A. alpina* significantly increased by 255% and 175%, respectively. Under S, Na_2_S, and Na_2_SO_4_ treatments, the lead content in the root and shoot organelles of *A. alpina* significantly reduced by 30%, 45%, and 63%, and by 22%, 59%, and 74%, respectively (Figure 2). In addition, the soluble components in the root significantly reduced by 24%, 42%, and 59% in sequence, and the soluble components in the shoot significantly increased by 74%, 138%, and 246% in sequence.

From Table 4, it can be seen that different forms and concentrations of sulfur increased the bio-accumulation factor (BCF) and translation factor (TF) of *A. alpina.* The BCF increased by 21% to 111% and the translocation coefficient increased by 33% to 181% compared to CK after treatment with S, S^2−^, and SO_4_^2−^, with the largest increase in the BCF and TF occurring when the Na_2_SO_4_ concentration was 150 mg·kg^−1^. The effects of different forms of sulfur on the enrichment and transport of lead in *A. alpina* were Na_2_SO_4_ > Na_2_S > S.

### 2.4. Correlation of Sulfur Content and Lead Content in Subcellular Parts of A. alpina

It can be seen from Table 5 that there is a significant positive correlation between the sulfur content of S^0^ and Na_2_SO_4_ under the treatments of 75 mg·kg^−1^ and 150 mg·kg^−1^ and the lead content of each subcellular part of *A. alpina* (*p* < 0.05). Under 150 mg·kg^−1^ treatment, Na_2_S had a significant positive correlation with the cell wall and organelles of the *A. alpine* root. It showed that the sulfur in *A. alpina* is closely related to the absorption of lead by the roots. Except for Na_2_SO_4_ at 75 mg·kg^−1^ and 150 mg·kg^−1^, there was a significant positive correlation between the sulfur content and the shoot cell wall of *A. alpina*. However, there was no significant correlation between the sulfur content and lead content in other shoot subcellular parts.

## 3. Discussion

Elemental sulfur cannot be directly absorbed and utilized by plants. It must be oxidized into sulfate in the soil through the S^0^-S_2_O_3_^2−^-S_4_O_6_^2−^-SO_4_^2−^ pathways to be absorbed by the plant [21]. The addition of different forms and concentrations of sulfur from external sources lowers the soil pH, and the circulation of sulfur enhances the activity of microorganisms in the soil [22], significantly increasing the biomass of the plant’s shoot and root [23]. Amounts of 50–100 mg·kg^−1^ Na_2_SO_4_ significantly promoted an increase in the shoot biomass of *A. alpina* [20]. In this study, different concentrations of Na_2_S treatment under Pb stress significantly increased the underground biomass of *A. alpina*. Na_2_S treatment at 150 mg·kg^−1^ had a significant effect on the shoot biomass. This is consistent with the significant increase in the *Robinia pseudoacacia* root system and biomass with Na_2_S treatment [24]. Na_2_SO_4_ treatment significantly increased the root and shoot biomass of *A. alpina*. Conversely, the effects of different concentrations of Na_2_SO_4_ on the shoot were not significant. It is speculated that the oxidation of Na_2_S-reducing substances in the rhizosphere affects the microenvironment and Eh (soil redox potential) of the rhizosphere soil, which in turn affects the activity of lead. The presence of SO_4_^2−^ can reduce the excessive heavy metals toxicity [25]; SO_4_^2−^ in the soil accumulates on the root surface of plants, which affects the adsorption, desorption, absorption, and antagonism of lead ions, which affects the bioavailability of lead. In this paper, Na_2_SO_4_ treatment significantly increased the biomass of *A. alpina*, and the root-to-shoot ratio of the three forms of sulfur treatment increased compared with the CK.

Cell wall fixation of Pb is important for plant detoxification, and the cell wall is the first plant cell structure to come into contact with heavy metals; the protoplasts can be protected from Pb toxicity [26,27]. Under Pb stress, both sulfur-containing compounds and Pb content were highest in the cellular fractions of the root in *A. alpina* when no sulfur treatment was added, reaching more than 50% in the organelles (Figure 1 and Figure 2). The sulfur content and Pb content in the root organelles gradually decreased after the addition of sulfur treatment; conversely, the proportion of sulfur-containing compounds in the cell wall increased, and the sulfur content and Pb content in the cell wall gradually increased with increasing levels of sulfur application. Under the sulfur treatment of 150 mg/Kg S, Na_2_S and Na_2_SO_4_, the root Pb content in the cell wall increased by 14% to 42% compared to CK (Figure 2), and a significant correlation (*p* < 0.05) between Pb content and cell wall and organelle sulfur content was observed (Table 5). In general, the sulfur application treatment increased the fixation of heavy metals in the cell wall of the root and shoot. The cell wall provides a large number of carboxyl, hydroxyl, amino and other functional groups, which provide a large number of binding sites so that they can interact with metal ions to form complexes and anchor them to the cell wall, restricting the transport of metal ions across the membrane and reducing transport to organelles [28]. In addition, the cell wall contains a lot of pectin and polysaccharide substances, and Pb stress induces an increase in pectin and polysaccharide content and their enzymatic activities, thereby promoting the uptake and accumulation of Pb by the cell wall [29].

As a hyperaccumulator, the shoot translocation of excess heavy metals from roots is an important accumulation mechanism [30]. The present study revealed that different forms and concentrations of sulfur treatment increased the BCF and facilitated the translocation of heavy metals from roots to shoot in *A. alpina*, all with a TF of greater than 1 (Table 4). It is generally agreed that xylem transport is a key process in the movement of metal ions from the subsurface to the shoot [31]. Some of the heavy metals in the xylem are bound to organic acids and amino acids, and the sulphate that the plant derives from the soil will be transferred through the xylem to the ectoplasmic continuum and coplasm; some of the heavy metals in this process will be bound to sulfur-containing compounds, and as the sulphate enters the plant organs or tissues, the sulphate in the plastids will be reduced or stored in the vacuolar. The structure of the vacuolar is disrupted during grinding, so the soluble component of the cell consists mainly of cytoplasm and vacuolar [12]. The S supply increases the vesicular isolation of Pb; isolating Pb into the vacuolar increases the Pb content of the soluble component (Figure 2), thus reducing the toxicity of Pb in the root. This is inconsistent with Das (2020), who suggested that exogenous sulfur not only increased the expression of root sulfur states and sulfate transporters (MsSULRT1;2 and MsSULTR1;3), but also reduced shoot Cd concentrations by increasing Cd content in root tissues. Under Cd stress, the exogenous sulfur-induced elevation of GSH in *alfalfa* root allows Pcs to bind to excess Cd, thereby reducing the toxicity of shoot Cd [13]. Under Cd stress, sulfur addition significantly reduced the content of hydrogen peroxide (CAT) and malondialdehyde (MDA) in plant leaves, and increased the activity of key enzymes in the ASA-GSH cycle and the content of antioxidant substances and phytoalexins, thus chelating excess cadmium in *Brassica juncea* and promoting the transport of cadmium from the root system to the shoot [3]. In the present study, the addition of Na_2_SO_4_ treatment significantly increased lead and sulfur content in the shoot cell walls of *A. alpina* by 2.75-fold and 4.58-fold and significantly reduced organelles by 3.77-fold and 9.27-fold, indicating that sulfur application enhanced the curing of lead in the cell walls of super-enriched plants in both root and shoot, and that vacuolar zone compartmentalization was the main way in which shoot sulfur enhanced lead tolerance in *A. alpina*.

It is speculated that a part of S^0^ and Na_2_S directly reacts with lead in the soil to form PbS (lead–sulfur combinations), which affects the bioavailability of lead in *A. alpina*, and part of the process that affects the microbial activity through sulfur redox reactions changes the physical and chemical properties of soil, such as pH and Eh (redox potential), and indirectly affects the behavior of lead [32]. *A. alpina* directly absorbs the SO_4_^2–^ in Na_2_SO_4_ to generate sulfur-containing functional groups that combine with lead to form high-affinity and stable compounds; this enhances the lead tolerance of *A. alpina.* Regarding how different forms of sulfur affect the characteristics of lead enrichment in *A. alpina*, it is necessary to use the near-side X-ray absorption fine structure absorption spectrometer to understand the geometric configuration of sulfur and lead atoms and reveal how sulfur regulates lead absorption and enrichment in plants.

## 4. Materials and Methods

### 4.1. Experimental Design and Materials

The test soil was obtained from an abandoned slag pile in Sanduoduo Village, Zhehai Town, Huize County, Yunnan Province (elevation 2463~2516 m above sea level, E: 103°03′~103°55′, N: 25°48′~28°38′). The collected soil was 0~20 cm tillage soil (Umbric, WRB) of corn-planted farmland near the slag heap. After air drying (laboratory temperature and relative humidity are 26–28 °C, 45–65%), the soil was passed through a 2 mm sieve, accurately weighed (5.0 kg), and placed in plastic pots (length 44 cm × width 15 cm × height 19 cm) for later use. The physical and chemical properties of the soil are shown in Table 6.

*A. alpina* seeds were sown in a seedbed with the substrate. After sprouting 1–2 leaves, seedlings with good growth and a uniform size were transplanted for the pot experiments. The planting method was to set 2 plants in one hole and 18 plants in 9 holes in each pot. The plants were watered with 1/2 Hoagland nutrient solution once every two days. Samples were collected for the experiment 90 days later.

The sulfur concentration was determined based on our previous studies, with the effect of sulfur nutrition on lead accumulation in *A. alpina* [33]. The concentrations of the three different sulfur forms (S^0^, Na_2_S, and Na_2_SO_4_) were set as 0 mg·kg^−1^, 75 mg·kg^−1^, and 150 mg·kg^−1^ and mixed with the appropriate weight of soil in pots. Each treatment was set with three parallel pots, with a total of 21 pots. The soil was sprayed with 500 mL of different concentrations and forms of sulfur, air dried, and re-mixed to speed up the homogenization process for 20 d.

### 4.2. Indicator Determination

#### 4.2.1. Soil Determination of Physical and Chemical Properties

The pH was determined using a pH meter and 1:2.5 (m/v) soil/water mixture, which was shaken for 30 min at room temperature and left for 5 h. In total, 0.1 g of 2 mm air-dried soil was weighted and placed in a 150 mL triangular flask. The soil with 5 mL HNO_3_-HClO_4_ was heated and digested with an adjustable electric heating plate until it was clear, transferred to a 50 mL volumetric flask, and the lead content was determined by an atomic absorption spectrophotometer (TAS-990, Agilent, Santa Clara, CA, USA). A coupled plasma emission spectrometer (ICAP 6300, Thermo Elemental, Waltham, MA, USA) was used to determine the available sulfur content; the organic matter was determined volumetrically with potassium dichromate; the available phosphorus was determined using a spectrophotometer (700 nm) (Thermo Scientific, Evolution260) after 0.5 M NaHCO_3_ leaching; the available nitrogen was determined by using the alkaline diffusion method; the available potassium was determined with flame photometry using 1 M NH_4_OAc leaching [34]; and cation exchange capacity was determined with a spectrophotometer using Co(NH_3_)Cl_3_ leaching (475 nm) [35].

#### 4.2.2. Determination of Biomass

The soil was brought to a semi-humid state with water, and the whole plant was pulled out from the soil. Plant roots were quickly rinsed with tap water to remove impurities attached to the roots and then rinsed with deionized water. The plant surface moisture was absorbed using filter paper and then evenly divided into two parts. One was deactivated at 105 °C for 30 min and then dried at 70 °C to a constant weight. The dry matter was weighed. Another fresh sample was kept to measure the subcellular sulfur and lead content.

#### 4.2.3. Determination of Subcellular Sulfur and Lead Content

Differential centrifugation was used to separate the subcellular components. The underground and aboveground parts of the prepared *A. alpina* fresh samples were separated. Exactly 1.00 g of fresh samples in a mortar was weighed. Pre-cooled subcellular extract (DTT: 1 mmol·L^−1^, sucrose: 0.25 mol·L^−1^, Tris-HCl: 50 mmol·L^−1^, and pH = 7.4) (10 mL), sucrose, and Tris-HCl were added to create an environment similar to that inside plant cells. DTT was added as an antioxidant, plant cells were broken using the low-temperature physical fragmentation method, and they were quickly placed in a mortar. They were then ground in an ice bath to a homogenate shape and transferred to a 50 mL centrifuge tube. Because of the lightness of the cell wall fraction, the precipitate was obtained by differential centrifugation at 1000 r·min^−1^ for 10 min. The supernatant was then removed, and the precipitate contained the cell wall component. The removed supernatant was centrifuged in a high-speed refrigerated centrifuge (TGL-20M, Thermo) at 12,000 r.min^−1^, at 4 °C, and for 45 min. The supernatant is the soluble fraction of the cell, and the precipitate is the organelle component. The sulfur and lead content measurement method are the same as that described in Section 4.2.1.

#### 4.2.4. Determination of Total Lead Content in the Plant and Soil

The Pb content of the roots and shoots are digested with HNO_3_-HC1O_4_ (*v*:*v* = 1:4) and determined using atomic absorption spectrophotometry (TF-4520, Shanghai Precision & Scientific Instrument Co., Ltd., Shanghai, China). Similarly, the total Pb content of the soil was digested with HNO_3_-H_2_O_2_ (*v*:*v* = 1:4) and determined using atomic absorption spectrophotometry [33].

### 4.3. Statistical Analysis

The data were collected and analyzed using Microsoft Excel 2010 (Redmond, WA, USA). One-way analysis of variance (one-way ANOVA) and a significance test (Duncan’s method, *p =* 0.05) were carried out using IBM SPSS Statistics 22 (Chicago, IL, USA). The data obtained are expressed as mean ± standard deviation (n = 3); figures were drawn using Origin 9.1.

The bio-accumulation factor and translocation factor of heavy metals in plants were calculated as follows:

$$\text{Bio-accumulation}\;\mathrm{factor}\;\left(BCF\right)=\frac{\mathrm{Heavy}\;\mathrm{metal}\;\mathrm{concentrations}\;\mathrm{in}\;\mathrm{shoots}/g}{\mathrm{Heavy}\;\mathrm{metal}\;\mathrm{concentrations}\;\mathrm{in}\;\mathrm{soil}/g}$$

$$\mathrm{Translocation}\;\mathrm{factor}\;\left(TF\right)=\frac{\mathrm{Heavy}\;\mathrm{metal}\;\mathrm{concentrations}\;\mathrm{in}\;\mathrm{shoots}/g}{\mathrm{Heavy}\;\mathrm{metal}\;\mathrm{concentrations}\;\mathrm{in}\;\mathrm{roots}/g}$$

## 5. Conclusions

In the present study, exogenous S^0^ application had no significant effect on the root-to-shoot ratio or the shoot and root dry weight of *A. alpina* under Pb stress. With increasing concentration, Na_2_S and Na_2_SO_4_ application promoted the growth of *A. alpina* and significantly increased the sulfur content in the cell wall of both root and shoot. BCF and TF increased, which promoted the translocation and accumulation of Pb. The correlation between the sulfur and Pb contents of subcellular fractions was found: there is a positive correlation between the sulfur and Pb contents of root and shoot cell walls. This suggests that the sulfur application reduced the transmembrane transport of heavy metals and that Pb fixation by the cell wall was the main way to reduce heavy metal toxicity in the root and shoot parts of *A. alpina*. In addition, sulfur application increased Pb content in the shoot soluble fraction, which may be a result of the soluble fraction including the vacuoles being the main site of Pb accumulation in the shoot of hyperaccumulators. In summary, these findings provide a reliable basis for the mechanism of Pb enrichment in *A. alpina* and other hyperaccumulators and for measures to reduce the heavy metal pollution of the environment by exogenous sulfur application.

## Figures and Tables

**Table 1:** Effects of different forms of sulfur on the biomass of *A. alpina* under lead stress.

Sulfur Forms	Treatment Concentration /mg·kg^−1^	Root /g·pot^−1^	Shoot /g·pot^−1^	Ratio of Root Biomass to Shoot Biomass
CK	0	0.17 ± 0.06 d	0.57 ± 0.10 d	0.30
S	75	0.20 ± 0.05 d	0.70 ± 0.06 d	0.29
	150	0.24 ± 0.03 d	0.58 ± 0.07 cd	0.41
Na_2_S	75	0.32 ± 0.03 c	0.71 ± 0.02 cd	0.45
	150	0.40 ± 0.05 b	0.77 ± 0.06 c	0.53
Na_2_SO_4_	75	0.52 ± 0.03 a	0.91 ± 0.04 b	0.57
	150	0.57 ± 0.04 a	1.16 ± 0.18 a	0.49

Note: CK was the non-treatment control, S was the S^0^ treatment, Na_2_S was the Na_2_S treatment, and Na_2_SO_4_ was the Na_2_SO_4_ treatment. The data in the table show the mean ± standard deviation of three replicates, and the different lowercase letters indicate significant differences among treatments (*p* < 0.05), respectively.

**Table 2:** Effects of different forms of sulfur on the sulfur contents in different subcellular components of *A. alpina*.

Treatment		Root/g·kg^−1^			Shoot/g·kg^−1^		
Sulfur Forms	Concentration /mg·kg^−1^	Cell Wall	Organelle	Soluble Fraction	Cell Wall	Organelle	Soluble Fraction
CK	0	1.03 ± 0.06 d	2.28 ± 0.56 a	0.12 ± 0.13 e	0.79 ± 0.16 d	3.34 ± 0.23 a	1.31 ± 0.07 e
S	75	1.74 ± 0.09 cd	1.24 ± 0.14 b	0.14 ± 0.08 e	1.03 ± 0.12 cd	1.71 ± 0.49 b	1.54 ± 0.11 de
	150	1.30 ± 0.05 d	0.95 ± 0.07 bc	0.17 ± 0.09 de	1.07 ± 0.11 cd	0.86 ± 0.13 c	1.87 ± 0.09 d
Na_2_S	75	1.68 ± 0.27 cd	0.80 ± 0.04 bc	0.22 ± 0.21 d	1.39 ± 0.19 c	0.67 ± 0.07 cd	2.45 ± 0.35 c
	150	2.22 ± 0.19 c	0.74 ± 0.06 bc	0.34 ± 0.37 c	1.44 ± 0.09 c	0.53 ± 0.03 cd	2.49 ± 0.45 c
Na_2_SO_4_	75	3.04 ± 0.48 b	0.48 ± 0.08 c	0.47 ± 0.20 b	2.41 ± 0.25 b	0.46 ± 0.02 cd	3.34 ± 0.23 b
	150	3.76 ± 0.32 a	0.39 ± 0.05 c	0.69 ± 0.85 a	3.62 ± 0.44 a	0.36 ± 0.04 d	4.39 ± 0.42 a

Note: CK was the non-treatment control, S was the S^0^ treatment, Na_2_S was the Na_2_S treatment, and Na_2_SO_4_ was the Na_2_SO_4_ treatment. The data in the table show the mean ± standard deviation of three replicates, and the different lowercase letters indicate significant differences among treatments, (*p* < 0.05), respectively.

**Table 3:** Effects of different forms of sulfur on the subcellular lead content of *A. alpina*.

Treatment		Root/mg·kg^−1^			Shoot/mg·kg^−1^		
Sulfur Form	Concentration /mg·kg^−1^	Cell Wall	Organelle	Soluble Fraction	Cell Wall	Organelle	Soluble Fraction
CK	0	87.39 ± 6.6 d	244.66 ± 33.6 a	457.65 ± 30.5 a	297.29 ± 39.3 d	256.12 ± 27.8 a	192.05 ± 46.3 f
S	75	137.90 ± 21.7 c	174.89 ± 28.3 b	406.93 ± 18.5 ab	381.42 ± 24.2 cd	260.02 ± 24.2 a	260.75 ± 33.0 ef
	150	173.99 ± 27.3 c	170.61 ± 16.6 b	349.73 ± 28.3 b	471.15 ± 82.8 c	199.28 ± 23.3 b	334.71 ± 43.3 de
Na_2_S	75	133.61 ± 17.8 c	142.72 ± 52.4 bc	263.92 ± 38.8 c	634.60 ± 52.0 b	144.05 ± 13.7 c	374.52 ± 26.5 d
	150	175.52 ± 20.6 c	134.36 ± 14.1 c	277.19 ± 55.6 c	706.78 ± 19.4 ab	104.58 ± 9.8 d	458.35 ± 34.1 c
Na_2_SO_4_	75	241.33 ± 27.9 b	134.18 ± 74.3 c	218.64 ± 31.8 cd	798.60 ± 67.2 a	80.17 ± 12.2 de	539.99 ± 56.0 b
	150	309.92 ± 38.0 a	89.85 ± 14.8 d	185.70 ± 17.5 d	817.66 ± 101.1 a	67.80 ± 5.8 e	664.89 ± 54.9 a

Note: CK was the non-treatment control, S was the S^0^ treatment, Na_2_S was the Na_2_S treatment, and Na_2_SO_4_ was the Na_2_SO_4_ treatment. The data in the table show the mean ± standard deviation of three replicates, and the different lowercase letters indicate significant differences among treatments, (*p* < 0.05), respectively.

**Table 4:** Effects of different forms of sulfur on the characteristics of lead accumulation in *A. alpina*.

Treatment		Root /mg·kg^−1^	Shoot /mg·kg^−1^	BCF	TF
Sulfur Forms	Concentration /mg·kg^−1^				
CK	0	789.7	745.46	0.28	0.94
S	75	719.72	902.19	0.34	1.25
	150	694.33	1005.14	0.38	1.44
Na_2_S	75	540.25	1153.17	0.44	2.13
	150	587.07	1269.71	0.48	2.16
Na_2_SO_4_	75	594.15	1418.76	0.54	2.38
	150	585.47	1550.35	0.59	2.64

Note: CK was the non-treatment control, S was the S^0^ treatment, Na_2_S was the Na_2_S treatment, and Na_2_SO_4_ was the Na_2_SO_4_ treatment. BCF was bioconcentration factor and TF was translocation factor.

**Table 5:** Correlation between lead and sulfur contents of subcellular in *A. alpina*.

Treatment		Root			Shoot		
Sulfur Forms	Concentration /mg·kg^−1^	Cell Wall	Organelle	Soluble Fraction	Cell Wall	Organelle	Soluble Fraction
CK	0	0.751	0.692	0.633	0.512	0.044	0.479
S	75	0.968 *	0.971 *	0.994 *	0.281	0.455	0.822
	150	0.997 *	0.997 *	0.982 *	0.929	0.436	0.651
Na_2_S	75	0.671	0.835	0.967 *	0.422	0.536	0.178
	150	0.996 *	0.956 *	0.864	0.649	0.195	0.112
Na_2_SO_4_	75	0.975 *	0.984 *	0.989 *	0.999 *	0.517	0.495
	150	0.991 *	0.973 *	0.962 *	0.964 *	0.197	0.728

Note: CK was the non-treatment control, S was the S^0^ treatment, Na_2_S was the Na_2_S treatment, and Na_2_SO_4_ was the Na_2_SO_4_ treatment. ‘*’ Indicates significant correlation (*p* < 0.05).

**Table 6:** Physical and chemical properties of soil.

pH	Available Sulfur /mg·kg^−1^	Pb /mg·kg^−1^	SOM /g·kg^1^	AP /mg·kg^−1^	AN /mg·kg^−1^	AK /mg·kg^−1^	CEC /cmol·kg^−1^
6.25	10.67	2618.74	16.87	12.08	116.34	28.77	16.45

Note: pH was potential of hydrogen, Pb was lead content, SOM was organic matter, AP was available phosphorus, AN was available nitrogen, AK was available potassium, and CEC was cation exchange capacity.

## Data Availability

The original contributions presented in the study are included in the article. Further inquiries can be directed to the corresponding author/s.
